# Association of serum vitamin D concentrations with dietary patterns in children and adolescents

**DOI:** 10.1186/s12937-018-0365-7

**Published:** 2018-06-04

**Authors:** Vijay Ganji, Bernadette Martineau, William Edmund Van Fleit

**Affiliations:** 1 Human Nutrition Department, College of Health Sciences, Qatar University, Doha, Qatar; 2 Children Healthcare of Atlanta, Emory University, Atlanta, GA USA; 3 Georgia State University, Atlanta, GA USA

**Keywords:** Dietary patterns, NHANES, Serum 25 hydroxyvitamin D, US children, Vitamin D

## Abstract

**Background:**

Because children have been advised on the dangers of sun exposure, diet is an important contributor of serum 25 hydroxyvitamin D [25(OH)D] concentrations. Aim of this study was to determine whether serum 25(OH)D concentrations were associated with any specific dietary patterns in US children.

**Methods:**

Data from 2 cycles of National Health and Nutrition Examination Survey (NHANES) 2003–2004 and 2005–2006 for individuals aged 2 to ≤19 y, were used to study relation between dietary patterns and serum 25(OH)D. We derived 2 major dietary patterns based on the food frequency questionnaire data. These were labeled as High-Fat-Low-Vegetable Dietary (HFLVD) pattern and Prudent Dietary (PD) pattern.

**Results:**

In multivariate adjusted analysis, there was no significant relationship between serum 25(OH)D concentrations and tertiles of HFLVD and PD dietary pattern scores in all subjects, boys, and girls. When dietary patterns scores were used as a continuous variable in adjusted analysis, children (all) with higher PD contribution scores to overall diet showed a significant positive relation with serum 25(OH)D (β = 59.1, *P* = 0.017). When data were stratified by sex, a significant positive relation was observed in girls between serum 25(OH)D concentration and PD pattern scores (β = 82.1, *P* = 0.015). A significant negative relation was observed in girls between serum 25(OH)D and HFLVD pattern scores (β = − 88.5, *P* = 0.016).

**Conclusion:**

Overall, serum 25(OH)D were associated with PD pattern but not with HFLVD pattern in US children. In public health perspective, it is important to encourage children, especially girls who are consuming HFLVD pattern to shift to healthier diet.

## Background

A classical function of vitamin D is to regulate extracellular calcium and phosphorus. Current evidence suggests that vitamin D may play a role in various non-bone diseases such as autoimmune disease [1, 2], cardiovascular disease [3, 4], type-2 diabetes mellitus (T2DM) [5], depression [6], and cancer [7]. Dietary vitamin D sources include oily fish such as salmon, mackerel, bluefish, sardines and tuna, shiitake mushrooms (fresh or sundried), and egg yolks. Fortified dietary sources of vitamin D include milk, orange juice, infant formulas, yogurts, butter, margarine, cheeses, and breakfast cereals [8].

Serum 25-hydroxyvitamin D [25(OH)D] is a commonly used marker of vitamin D nutritional status. Hypovitaminosis D is a widespread problem in the US, specifically in children and adolescents [9–12]. Prevention of suboptimal vitamin D status in childhood may reduce future adverse health conditions. The contribution of dietary sources to vitamin D status is not clearly known in children. Some studies have shown that dietary intake of certain vitamin D rich foods had a significant positive influence on serum 25(OH)D concentrations [13, 14], whereas other studies have shown that vitamin D intake did not affect serum 25(OH)D concentrations [15]. While sunlight exposure is the major source of circulating serum 25(OH)D [16], children and adolescents have been advised on the dangers of sun exposure [17] and are exposed to increased use of sunscreen lotions and time spent indoors which has likely contributed to the increasing prevalence of low vitamin D status [18]. Therefore, diet is an important contributor of circulating serum 25(OH)D in the absence of or in the presence of reduced sunlight exposure.

To our knowledge, no data are available on the relation between dietary patterns and serum 25(OH)D in US children and adolescents. Studies that have looked at diet and vitamin D status have addressed associations between individual food sources such as fortified milk or fatty fish. However, people consume a variety of foods in combination [9]. Dietary pattern analysis, an alternative approach to traditional single nutrient epidemiology, takes into account all nutrient interactions and allows for a more comprehensive approach to study the relation between disease and dietary intake [19]. Therefore, the objective of this study was to determine whether serum 25(OH)D concentrations were associated with any specific dietary patterns in US children and adolescents using assay-adjusted data from the National Health and Nutrition Examination Survey (NHANES) 2003–2006.

## Methods

### Brief NHANES survey methods

The National Center for Health Statistics (NCHS) conducts large, nationally representative, sample surveys known as NHANES on the noninstitutionalized US civilian population. A sample representative of individuals aged > 2 months was selected by using a stratified, multistage, probability sample survey design. Beginning in 1999, NHANESs were conducted as annual surveys and data are released in 2-y cycles for public use. Certain subgroups including low-income persons, adolescents, persons aged ≥60 y, non-Hispanic black (NHB), and Hispanic/Mexican American (H/MA) are oversampled to yield more reliable estimates for these specific groups. The detailed descriptions of the survey design and methodologies are described elsewhere [20].

NHANES 2003–2004 was conducted between January 2003 and December 2004 in 12,761 individuals [9643 were examined in the Mobile Examination Centers (MEC)] and NHANES 2005–2006 was conducted between January 2005 and December 2006 in 12,862 individuals (9950 were examined in the MECs). Participants were interviewed in their homes to gather information on demographic characteristics, diet, and health. Additional health data were collected during a medical examination conducted in MECs. At the MECs, a physical exam, blood and urine sample collection, and other diagnostic measurements were performed. All NHANES protocols were approved by the NCHS Ethics Review Board prior to data collection. Detailed description of these protocols is found elsewhere [21].

Households were randomly selected and all members within the household were screened for demographic characteristics. One or more individuals within the household were then selected for sample based on age, sex, and race-ethnicity. NHANES 2003–2006 sample included individuals ≥2 mo old. Race-ethnicity was categorized as non-Hispanic white (NHW), NHB, H/MA, and Other. Participants self-reported their race-ethnicity status. Poverty income ratio (PIR) was calculated as the ratio of income to the family’s appropriate poverty threshold. To avoid damage to the MECs, examination data in the North were collected in spring/summer (May 1–October 31) and in South were collected in fall/winter (November 1–April 30). Data for body mass index (BMI) were obtained from the medical examination component of NHANES. Supplement users were defined based on participants who answered ‘yes’ to the question “Did you take supplements in the past 30 d?” Participants were asked about hours spent watching television, playing video games, and using the computer.

Blood samples were collected by venipuncture from participants in MECs according to standard protocols. Detailed specimen collection and processing methods have been previously reported [22, 23]. Serum 25(OH)D concentrations were analyzed and determined at the National Center for Environmental Health, Centers for Disease Control and Prevention using the Diasorin Radioimmunoassay (Stillwater, MN).

Periodically, NHANES data files are updated by the NCHS, replacing previous data files. In November 2010, an update occurred for serum 25(OH)D data because of changes and drifts in serum 25(OH)D assay over time. This was likely due to method variation that resulted from reagent and calibration lot-to-lot variation. The NCHS released a data advisory for vitamin D and recommended use of the assay-adjusted data by investigators rather than previously available unadjusted data. A detailed description of this data advisory for serum 25(OH)D is described elsewhere [24].

A 216-item food frequency questionnaire (FFQ) component was newly added to NHANES 2003–2004 and was used to gather information on the frequency of food consumption of participants over the past 12 months. The questionnaire was developed, tested, and validated by the National Institutes of Health, National Cancer Institute. Participants were asked the average number of times foods were consumed over the past 12 months and for certain types of foods, their seasonal intake were also gathered. Participants reported the number of times/d, wk., mo, or never that a food was consumed. All foods’ frequency of consumption was standardized to a monthly intake by using a conversion factor of 30.4 d/mo as this is the number of days in an average month. Frequency of consumption was collected for dairy products, meat, fish and seafood, poultry, eggs, fruits and juices, vegetables, grains and legumes, snacks and sweets, beverages, and added fats. Those participants who did not answer the FFQ were excluded from this study.

### Study sample

**Fig. 1:**
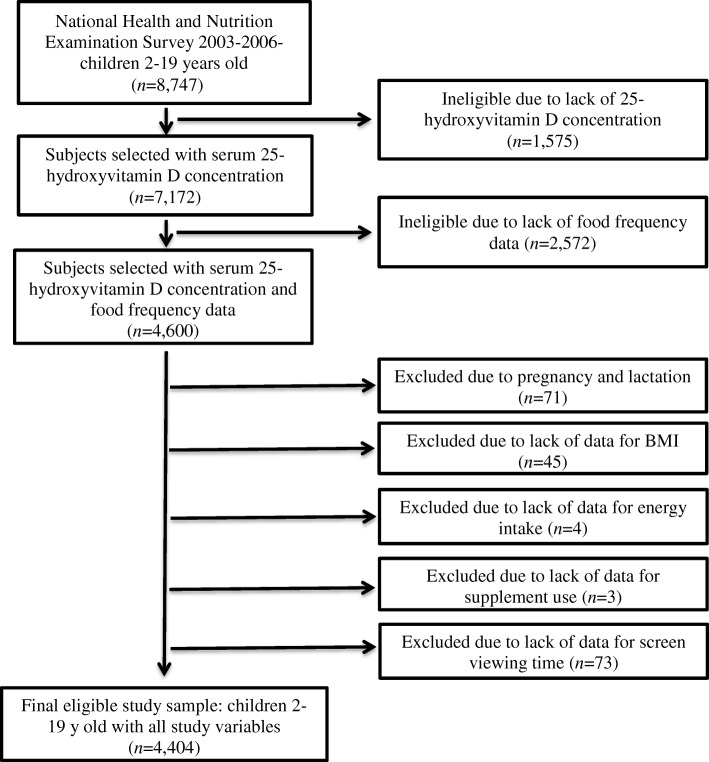
Study sample derivation after removing subjects for missing data for main variables and confounding variables sequentially (weighted *n* = 60,274,698)

Two cycles of NHANES 2003–2004 and 2005–2006 were used in this study. Although serum 25(OH)D concentrations are available publically in NHANES 2001–2002, this survey was not included in this current study because FFQ data were not collected. Data on children between ages 2 to ≤19 y from NHANES 2003–2004 and 2005–2006 were concatenated into one master analytic database, NHANES 2003–2006 (*n* = 8747). Subjects with serum 25(OH)D concentration data were then selected to include in this study (*n* = 7172). Of the remaining 7172 participants, children < 2 y old were excluded from the data analysis due to lack of completed FFQ (*n =* 2572). Of the remaining 4600 children, 71 were excluded because they reported that they were lactating or pregnant, 45 were excluded due to lack of BMI measurement, 4 were excluded due to lack of calorie intake data, 3 were excluded due to lack of supplement use data, and 73 were excluded due to lack of screen viewing response. Thus, the final analytic sample consisted of 4404 children and adolescents (Fig. 1).

### Study variables

In this study, the foods from the FFQ were categorized into 30 food groups. These 30 food groups were low-fat and high-fat dairy products, dairy alternatives, fish and other seafood, eggs, meat, processed meat, poultry, creamed soups, other soups, pizza, mixed foods, cereals, refined grains, whole grains, nuts, legumes, tomatoes, cruciferous, starchy, and other vegetables, fruit, fruit juices, snacks and sweets, butter and margarine, other fats, added sugars, coffee/tea, energy drinks (high or low), and alcohol (Table 1). Foods were categorized based on nutrient profiles or culinary use and were grouped similar to those used in other studies [25]. Frequency of dietary intake of these 30 food groups for each individual was used to identify major dietary patterns. Age, sex, race-ethnicity, BMI, PIR, time of examination, use of supplements, energy intake, and screen use hours were considered as potential confounding variables as these are known to affect serum 25(OH)D concentrations [18, 26]. Participants were categorized into 2–3 y, 4–8 y, 9–13 y, and 14–19 y old age groups. BMI was categorized as normal weight (<85th percentile) and overweight and obese (≥85th percentile) for age and sex. PIR was categorized as below poverty (< 1.0), middle income (1.0–2.5), higher income (> 2.5), and not reported. Combined television, computer, and video game use hours were categorized as ≤2 h, 3–4 h, or > 4 h/d. Smoking status and alcohol intake variables were also considered as potential confounding variables. However, smoking-related questions were only asked to children aged ≥12 y and alcohol-related questions were asked to adults aged ≥20 y; therefore, both were dropped from the analysis.

**Table 1:** Food groups used in the dietary pattern analysis: NHANES 2003-2006^a^

Food Groups^b^	Foods from Food-Frequency
	Questionnaire^c^
Low-fat dairy	1, 2%, skim, nonfat, and evaporated milk; yogurt/frozen, low-fat cheese,and low-fat sour cream
High-fat dairy	Whole milk, cream, ice cream, pudding, cottage cheese, cheese, and sour cream
Dairy alternative	Soy, rice, and other milk; non-dairy creamer, and meal replacement beverage
Fish and other seafood	Oysters, clams, and shellfish; fish: fillets, sticks, tuna, salmon, and raw fish sushi
Eggs	Egg whites, whole egg, egg substitute, and egg salad
Meat	Beef, steak, roasts, hamburger, pork, ribs, and ham
Processed Meat	Bacon, Canadian bacon, sausage, hot dogs, luncheon meats, liver, and Liverwurst
Poultry	Chicken, all types; and turkey
Creamed soup	Creamed soups, all types; and chowders
Other soup	Broth-based soups and bean soups
Pizza	Pizza, all types
Mixed dishes	Casseroles, lasagna, macaroni and cheese, and chili
Cereal	Oatmeal, grits, and other cooked cereals; and cold cereal, all types
Refined grains	English muffin, bagel, roll, cracker, stuffing, cornbread, biscuit, pancake, waffle, pasta, and rice
Whole grains	Dark breads and rolls; brown rice, bulgur, cracked wheat and millet; and granola bars
Nuts	Peanuts, walnuts, and other nuts; seeds; and nut butters
Legumes	Pintos, kidney, blackeyed peas, lima, lentils, refried beans, baked beans, soybeans, and tofu
Starchy vegetables	White potatoes, french fries, and potato salad; squash, sweet potatoes, carrots, and yams
Tomatoes	Tomatoes, including fresh, tomato juice, and salsa
Cruciferous and green vegetables	Spinach, turnip, collard, chard, kale, broccoli, cabbage, cauliflower, Brussel sprouts, and lettuce
Other vegetables	Pickles, green beans, peas, peppers, onion, cucumber, corn, and mixed Vegetables
Fruit	Apples, pears, peaches, bananas,melons, strawberries, grapes, pineapple, and dried fruit
Fruit juices	Orange juice, grapefruit juice, apple juice, grape juice, and prune juice
Sweets and Snacks	Donuts, danish, cookie, brownie, cake, pie, cobbler, popcorn, pretzels, tortilla chips, and candy
Butter and Margarine	Butter and margarine, all types
Other fats	Olive oil, corn oil, canola oil, salad dressings, mayonnaise, and gravies
Condiments	Maple syrup, honey, jam, and jelly
Coffee/Tea	Coffee and tea, regular and decaffeinated
Energy drinks	Sodas and fruit drinks, including Hi-C, Kool-Aid, lemonade, and cranberry cocktail
Alcohol	Beer, wine, wine coolers, hard liquor, and mixed drinks

^a^*n* = 4404; weighted *n* = 60,274,698. NHANES 2003–2004 and 2005–2006 were combined into one master database, NHANES 2003–2006

^b^Foods consumed by survey participants were categorized into 30 food groups based on nutrient profiles or culinary use

^c^Food consumption data were collected using a 216-item qualitative Food Frequency Questionnaire

### Data analysis

Statistical analysis was performed using SAS statistical software (version 9.2, SAS Institute) as it is capable of handling the complex survey design of NHANES. The survey analysis procedures accounted for primary sampling unit, stratum, cluster, and observation weight in the calculation of variances used for interval estimation and hypothesis testing. The NOMCAR option was used in all analyses so that design variables with missing values are used in the domain analysis to estimate variances using Taylor series linearization method. Detailed guidelines on the sample weighting and the proper variance estimation procedures are outlined in the NHANES Analytic and Reporting Guidelines [20].

Factor analysis (principal component) was used to identify dietary patterns based on the frequency of dietary intake of the 30 predefined food groups. The PROC FACTOR procedure in SAS was used to conduct this analysis. The factors were rotated by orthogonal transformation to achieve a structure of independent factors with greater interpretability. The number of factors that were retained was determined based on an Eigenvalue (≥1.5), explained variance (≥5%), and Cattell scree plot. The remaining factors were considered the main dietary patterns and were labeled based on interpretation of the data. Factor loadings were derived for each of the 30 food groups across the extracted factors. For each dietary pattern, a factor score was calculated for each participant by combining the frequency of dietary intake of the food groups weighted by their factor loadings. Dietary pattern scores were then stratified into tertiles (low, medium, and high) based on the factor scores for each dietary pattern.

Chi-square tests were used to identify associations between demographic, lifestyle, and health characteristics among the dietary pattern tertiles. Multivariate-adjusted regression analysis was used to determine the associations between serum 25(OH)D concentrations and dietary patterns. Associations were analyzed according to the participants’ factor scores for each dietary pattern divided into tertiles (low, medium, and high), and to the factor scores as a continuous variable. This analysis included sex, age, race-ethnicity, use of supplements, time of examination, BMI, PIR, and screen use hours as potential confounding variables. Variables found to be non-significant such as PIR and use of supplements were dropped from the model. Because previous studies found differences of serum 25(OH)D concentrations by sex [11, 26], the present analysis for the relation between serum 25(OH)D and dietary patterns was then stratified by sex. Univariate ANOVA was used to establish if serum 25(OH)D concentrations varied across dietary patterns for all subjects, boys, and girls in an unadjusted analysis. Analysis of covariance (ANCOVA) was utilized to establish if serum 25(OH)D concentrations varied across dietary patterns after adjusting for various confounding variables. Multiple comparisons among dietary patterns for serum 25(OH)D concentrations were made using independent unpaired t-tests with a Bonferroni correction. Serum 25(OH)D concentrations were presented as mean ± standard error (SE). Statistical significance was set at α = 0.05.

## Results

### General demographic characteristics of study sample

The sample consisted of 51.5% (*n* = 2154) boys and 48.5% (*n* = 2250) girls. Of the 4404 participants, 62.5% (*n* = 1293) were NHW, 15.3% (*n* = 1428) were NHB, and 13.3% (*n* = 1323) were H/MA. The participants were distributed across the age categories: 8.4% (*n* = 399) 2–3 y, 26.4% (*n* = 924) 4–8 y, 29.9% (*n* = 1282) 9–13 y, and 35.3% (*n* = 1799) 14–19 y olds. Of the study population, 34.1% (*n* = 1133) reported having taken a supplement 30 d prior to the completing the survey. The majority (61.1%, *n* = 2215) of the participants were examined in the summer. 84.8% (*n* = 3626) were classified as healthy weight and 15.2% (*n* = 778) as overweight and obese based on the BMI. The majority (49.9%, *n* = 1984) reported ≤2 h/d of television, computer, and video game usage, although 28.5% (*n* = 1296) reported between 3 and 4 h/d of usage and 21.6% (*n* = 1124) reported > 4 h/d of usage.

### Dietary patterns

Two major dietary patterns were identified based on the factor analysis from the 30 predefined food groups (Table 1). Higher positive factor loading scores are interpreted to contribute most to the factor score, and conversely, higher negative factor loading scores contribute least to the factor score. The 1st factor had heavy factor loading scores for meats, snacks and sweets, condiments, mixed dishes, pizza, processed meats, refined grains, high fat dairy, coffee/tea, poultry, starchy vegetables, and fish and other seafood. Thus, the 1st factor was labeled as the High-Fat-Low-Vegetable Dietary (HFLVD) pattern. This was the most dominant dietary food pattern in the population and explained largest variance in food intake. The 2nd factor had heavy factor loading scores for all vegetable groups, fruit, other fats, mixed dishes, fish and other seafood, tomatoes, and meats. Thus, the 2nd factor was labeled as the Prudent dietary (PD) pattern. The detailed factor loading matrixes are listed in Table 2.

**Table 2:** Factor loading matrix for dietary patterns in National Health and Nutrition Examination Survey (NHANES) 2003-2006^a,b^

Category^c^	Factor 1: HFLVD Pattern^d^	Factor 2: PD Pattern^d^
Cruciferous & green vegetables	0.118	0.705
Other vegetables	0.144	0.734
Tomatoes	0.172	0.464
Starchy vegetables	0.514	0.518
Fruit	0.199	0.676
Fruit juice	0.364	0.243
Nuts	0.228	0.409
Legumes	0.085	0.406
Fish & other seafood	0.505	0.479
Meat	0.645	0.457
Poultry	0.519	0.392
Processed Meat	0.580	0.346
Whole grains	−0.049	0.448
Refined grains	0.570	0.448
Cereals	0.084	0.347
Eggs	0.279	0.298
Low fat dairy	−0.136	0.413
High fat dairy	0.543	0.133
Dairy Alternative/Meal Replacement	0.203	0.082
Creamed soups	0.163	0.196
Other soups	0.299	0.415
Mixed dishes	0.605	0.523
Pizza	0.604	0.089
Snacks & sweets	0.635	0.338
Butter & Margarine	0.423	0.288
Other fats	0.353	0.525
Condiments	0.632	0.173
Energy drinks	0.513	−0.074
Alcohol	0.120	−0.018
Coffee/Tea	0.523	−0.078

^a^*n* = 4404; weighted, *n* = 60,274,698. Correlation coefficients

^b^Factor procedure, principal component analysis. Two factors with Eigenvalues ≥1.5 were rotated and extracted. Factors were labeled according to the foods found to have the highest correlation coefficients within each factor (dietary pattern). Positive factor scores indicate that those foods are more likely to be consumed in that dietary pattern. Lower negative scores indicate that those foods are least likely to be consumed in that dietary pattern

^c^Food categories were based on consumption data collected from a 216-item Food Frequency Questionnaire from NHANES 2003–2004 and 2005–2006. Individual foods were categorized into 30 food groups

^d^High-Fat-Low-Vegetable Dietary Pattern or Prudent Dietary Pattern

### Characteristics of study population by dietary pattern

The sample distribution by characteristics of the study population across tertiles of each dietary pattern is presented in Table 3. Proportion of persons belonging to NHW and H/MA race-ethnicities tended to decrease, while proportion of persons belonging to NHB tended to increase across the tertiles of HFLVD pattern scores (*P* < 0.001). Proportion of children in the 14–19 y old age group tended to increase from tertile 1 (30.1%) to tertile 3 (40.6%) (*P* = 0.003), while their proportion tended to decrease from tertile 1 (48.8%) to tertile 3 (26.8%) (*P* < 0.001). In both the HFLVD and PD patterns, the proportion of children in the lowest PIR category tended to increase from the lowest tertile to the highest tertile pattern scores. In the HFLVD pattern, the proportion of children who viewed ≤2 h of combined television, computer, and video games tended to decrease from the lowest tertile (59.8%) to the highest tertile (41.6%) pattern scores (*P* < 0.001), while they tended to increase from the lowest (42.7%) to the highest tertile (55.4%) pattern scores (*P* < 0.001).

**Table 3:** Sample distribution by demographic and health characteristics according to the tertiles of factor scores for dietary patterns: National Health and Nutrition Examination Survey (NHANES) 2003-2006^a, b^

Characteristic	HFLVD Pattern Score^c^(*n* = 4404)				PD Pattern Score^c^(n = 4404)			
	Low (<−0.003875)	Medium (−0.003875 to 0.000925)	High (> 0.000925)	*P* for Trend^d^	Low (< 0.004175)	Medium (− 0.004175 to 0.001912)	High (> 0.001912)	*P* for Trend^d^
N	1338	1465	1601		1523	1376	1505	
Sex								
Boys, %	48.6	51.8	54.2	0.165	48.7	55.8	50.1	0.098
Girls, %	51.4	48.2	45.8		51.3	44.2	49.9	
Race-ethnicity								
NHW, %	70.0	62.2	55.2	< 0.001	61.0	66.4	60.0	0.003
NHB, %	7.3	13.9	24.7		18.0	13.2	14.7	
H/MA, %	14.3	14.1	11.5		10.9	13.1	15.9	
Other, %	8.3	9.8	8.6		10.9	7.3	9.4	
Age								
2–3 y, %	9.6	8.9	6.7	0.003	5.3	8.9	11.0	< 0.001
4–8 y, %	25.8	26.6	26.8		18.6	30.0	30.4	
9–13 y, %	34.6	29.4	25.8		27.3	30.7	31.8	
14–19 y, %	30.1	35.1	40.6		48.8	30.4	26.8	
Poverty income ratio^e^								
< 1.0, %	16.7	19.6	28.4	< 0.001	20.4	20.0	24.3	0.001
1.0–2.5, %	33.0	31.9	32.0		32.5	29.8	34.7	
≥2.5, %	48.1	44.7	37.2		42.3	48.2	39.3	
Not reported, %	2.1	3.8	2.4		4.8	2.0	1.6	
Season of Examination^f^								
Fall/Winter, %	40.2	39.6	36.8	0.693	39.0	36.3	41.4	0.395
Spring/Summer, %	59.8	60.4	63.2		61.0	63.7	58.6	
Use of Supplements^g^								
Yes, %	38.4	35.3	28.5	0.003	27.0	36.4	38.9	< 0.001
No, %	61.6	64.7	71.5		73.0	63.6	61.1	
Body mass index								
<85th percentile, %	86.0	84.0	84.5	0.610	81.8	86.2	86.4	0.028
≥85th percentile, %	14.0	16.0	15.5		18.2	13.8	13.6	
Daily screen viewing^h^								
≤2 h, %	59.8	48.4	41.6	< 0.001	42.7	51.6	55.4	< 0.001
3–4 h, %	25.1	28.8	31.5		30.2	27.8	27.4	
> 4 h, %	15.2	22.8	26.9		27.0	20.6	17.3	

^a^*n* = 4404; weighted *n* = 60,274,698. NHANES 2003–2004 and 2005–2006 were combined into one master database, NHANES 2003–2006

^b^Dietary pattern scores were stratified into tertiles (low, medium, and high) based on factor scores for each dietary pattern

^c^High-Fat-Low-Vegetable Dietary pattern or Prudent Dietary pattern

^d^Significance determined by Rao-Scott chi-square test

^e^Ratio of income to the family’s appropriate poverty threshold. A ratio of < 1.0 is characterized as below poverty

^f^Data collected during May 1–October 31 (spring/summer) and November 1–April 30 (fall/winter)

^g^Participants who took supplements 30 days before survey was conducted

^h^Data collected on the combined hours of television, computer, and video games usage per day

Subjects in the low-intake group of the HFLVD pattern (*P* for trend = 0.003) and the high-intake group of the PD pattern (*P* for trend< 0.001) were more likely to have consumed supplements. Subjects in the low-intake group of the PD pattern had a higher BMI (*P* for trend < 0.001. Subjects in the high-intake group of the HFLVD pattern had higher combined usage of television, computer, and video games/d than those in the low-intake group (≤2 h/d) (*P* for trend < 0.0001). Subjects in the high-intake group of the PD pattern had lower combined usage of television, computer, and video games/d (*P* for trend < 0.0001).

### Relation between serum 25(OH)D and dietary patterns

The relation of mean serum 25(OH)D concentrations and dietary pattern according to the tertiles of factor scores are presented in Table 4. In the unadjusted regression analysis, serum 25(OH)D concentrations differed significantly across the tertiles of the HFLVD pattern scores (*P* = 0.003) and PD pattern scores (*P* = 0.012). Subjects who had low-intake HFLVD pattern scores had greater serum 25(OH)D concentrations compared to those with medium and high-intake pattern scores (+ 1.2 ± 0.1 ng/mL and + 2.5 ± 0.01 ng/mL, respectively). When the model was adjusted to confounding variables, mean serum 25(OH)D concentrations of the boys and girls with low, medium, and high-intake HFLVD or PD patterns scores did not differ significantly. Girls with medium and high PD pattern scores had slightly higher mean serum 25(OH)D concentrations compared to those with low pattern score (21.5 ± 0.5 vs. 19.9 ± 0.6 ng/mL; *P* = 0.064).

**Table 4:** Relation of serum 25(OH)D concentrations with dietary pattern scores: National Health and Nutrition Examination Survey (NHANES) 2003-2006^a,b^

HFLVD Pattern Score (*n* = 4404)^c, d^					PD Pattern Score (*n* = 4404)^c, d^			
	Low (<− 0.003875)	Medium (− 0.003875 to 0.000925)	High (> 0.000925)	*P*- value^e^	Low (< 0.004175)	Medium (− 0.004175 to 0.001912)	High (> 0.001912)	*P*- value^e^
	ng/mL	ng/mL	ng/mL		ng/mL	ng/mL	ng/mL	
Unadjusted analysis								
All subjects (*n* = 4404)	27.3 ± 0.5	26.1 ± 0.6	24.8 ± 0.7	0.003	24.7 ± 0.7	26.8 ± 0.5	26.7 ± 0.6	0.012
Boys (*n* = 2154)	27.4 ± 0.6	26.8 ± 0.7	25.8 ± 0.8	0.123	25.8 ± 0.7	26.9 ± 0.7	27.3 ± 0.7	0.151
Girls (*n* = 2250)	27.2 ± 0.7	25.2 ± 0.8	23.6 ± 0.8	0.003	23.6 ± 0.9	26.6 ± 0.6	26.1 ± 0.7	0.005
Adjusted analysis								
All subjects (*n* = 4404)^f^	22.1 ± 0.4	22.1 ± 0.3	21.7 ± 0.5	0.594	21.4 ± 0.5	22.1 ± 0.3	22.5 ± 0.4	0.195
Boys (*n* = 2154)^g^	22.9 ± 0.5	23.3 ± 0.4	23.1 ± 0.6	0.810	23.0 ± 0.5	22.9 ± 0.4	23.5 ± 0.5	0.370
Girls (*n* = 2250)^g^	21.4 ± 0.5	20.9 ± 0.5	20.4 ± 0.7	0.529	19.9 ± 0.6	21.5 ± 0.5	21.5 ± 0.5	0.064

^a^*n* = 4404; weighted *n* = 60,274,698. NHANES 2003–2004 and 2005–2006 were combined into one master database, NHANES 2003–2006. Regression analysis of dietary patterns scores and serum 25(OH)D concentrations

^b^Dietary pattern scores were stratified into tertiles (low, medium, and high) based on factor scores for each dietary pattern

^c^Mean ± standard error

^d^High-Fat-Low-Vegetable Dietary Pattern or Prudent Dietary Pattern

^e^Significance determined by F-test in analysis of variance for unadjusted analysis and in analysis of covariance for adjusted analysis

^f^Analysis was adjusted for sex, race-ethnicity, age, season of examination, body mass index, and daily screen viewing. Poverty income ratio, supplement use, and energy intake were not found significant in this model and therefore those variables dropped

^g^Analysis was adjusted for race-ethnicity, age, time of examination, body mass index, and daily screen viewing. Poverty income ratio, supplement use, and energy intake were not found significant in this model and therefore these variables were dropped

The relation between serum 25(OH)D concentrations and dietary pattern scores as a continuous variable is presented in Table 5. In unadjusted analysis, subjects with higher HFLVD contributions scores to overall diet showed a significant negative relation to serum 25(OH) concentrations (*β* = − 135.56; *P* < 0.001). In the same unadjusted analysis, subjects with higher PD contribution scores to overall diet did not show a significant association with serum vitamin D concentrations (*β* = − 57.1; *P* = 0.073). However when subjects were stratified into boys and girls, only girls showed significant relation with serum 25(OH)D concentrations for both dietary pattern scores. Girls with higher HFLVD contribution scores showed a significant negative relation (*β* = − 193; *P* < 0.001) and girls with higher PD contribution scores showed a significant positive association to serum 25(OH)D concentrations (*β* = 79.8; *P* = 0.035).

**Table 5:** Relation of serum 25(OH)D concentrations with dietary pattern scores: National Health and Nutrition Examination Survey (NHANES) 2003-2006^a,b^

	β^c^	Standardized β	SE for β^d^	*P*-value^e^
Unadjusted analysis				
HFLVD Pattern Score^f^				
All subjects	−135.6	−0.13	32.3	< 0.001
Boys	−81.4	−0.08	47.8	0.099
Girls	−193.0	−0.19	36.7	< 0.001
PD Pattern Score^f^				
All subjects	57.12	0.06	30.8	0.073
Boys	36.13	0.04	31.8	0.265
Girls	79.75	0.08	36.1	0.035
Adjusted analysis				
HFLVD Pattern Score^f^				
All subjects^g^	− 39.1	−0.04	26.6	0.153
Boys^h^	17.7	0.02	36.3	0.631
Girls^h^	− 88.5	−0.09	34.6	0.016
PD Pattern Score^f^				
All subjects^g^	59.1	0.06	23.5	0.017
Boys^h^	35.7	0.03	24.1	0.149
Girls^h^	82.1	0.08	31.8	0.015

^a^*n* = 4404; weighted *n* = 60,274,698. NHANES 2003–2004 and 2005–2006 were combined into one master database, NHANES 2001–2006

^b^Regression analysis using factor scores as continuous variable and dependent variable, serum 25(OH)D concentrations

^c^Multivariate regression coefficient

^d^Standard error for multivariate regression coefficient

^e^Significance between dietary patterns and serum 25(OH)D in the regression model

^f^High-Fat-Low-Vegetable Dietary pattern or Prudent Dietary pattern

^g^Analysis was adjusted for sex, race-ethnicity, age, season of examination, body mass index, and daily screen viewing. Poverty income ratio, supplement use, and energy intake were not found significant in this model, therefore those variables were dropped from the analysis

^h^Analysis was adjusted for race-ethnicity, age, season of examination, body mass index, and daily sun screen viewing. Poverty income ratio, supplement use, and energy intake were not found significant in this model, therefore those variables were dropped from the analysis

In the adjusted multivariate regression analysis, all subjects with higher PD contribution scores to overall diet showed a significant positive relation with serum 25(OH)D (*β* = 59.1; *P* = 0.017). Similarly, when subjects were stratified into boys and girls in the adjusted analysis, only girls showed a significant relation with serum 25(OH)D concentrations for both pattern scores. Girls with higher HFLVD contribution scores showed a significant negative relation (*β* = − 88.5; *P* = 0.016) and girls with higher PD contribution scores showed a significant positive association (*β* = 82.1; *P* = 0.015) to serum 25(OH)D concentrations.

## Discussion

To our knowledge, this is the most comprehensive study that investigated the relation between serum 25(OH)D concentrations and dietary patterns in children and adolescents in a nationally representative sample survey. Using factor analysis, we derived HFLVD and PD patterns. Serum 25(OH)D was significantly lower in HFLVD compared to PD pattern, and the highest serum 25(OH)D concentrations for all subjects were in those with low HFLVD or medium and high-intake PD patterns scores. In the multivariate adjusted analysis, a significant positive relation was found between PD pattern factor scores and serum 25(OH)D concentrations. When data were stratified by sex, a significant positive relation was observed in girls who consumed the PD dietary pattern and a significant negative relation was observed in girls who consumed the HFLVD dietary pattern.

Dietary patterns derived in this study were similar to the dietary patterns reported in the literature on US children [27]. Poti et al. [27] using the NHANES data derived Western and Prudent dietary patterns. Studies relating vitamin D intake with the vitamin D status have shown conflicting results [13, 15, 27]. We found that subjects with high PD pattern scores had significantly higher serum 25(OH) concentrations compared to those with HFLVD pattern scores. In contrast, Polish vegetarian children had 2-fold lower serum 25(OH)D concentrations than in their omnivorous counterparts [28]. While in this study those who consumed the PD pattern were not necessarily vegetarians, they did have higher factor scores for many similar type of foods found in a vegetarian diet and lesser factor scores for foods typical in an omnivorous diet. However Chan et al. [29] found no association between serum 25(OH)D and vegetarian status. Differences between studies may be due to differences in subject characteristics and confounding variables used in the statistical analysis.

The highest serum concentrations for all subjects were found in those with low-intake HFLVD scores or in those with medium- and high-intake PD pattern scores. In this analysis individuals were scored on each pattern, therefore a person’s diet would be represented by a combination of both factors. A high factor score from one dietary pattern does not necessarily mean a low factor score from the other dietary pattern for an individual. However, these results seem to suggest that the greatest serum 25(OH)D concentrations occurred in individuals who consumed a healthier type diet that had a higher emphasis on vegetables, fruits, and some emphasis on mixed dishes, fish, and meats. The differences seen in serum 25(OH)D concentrations may be due to the factors other than diet; because when the analysis was adjusted for confounding variables, the association between serum 25(OH)D concentrations and dietary patterns was no longer present. This was seen in other studies such that other factors such as race, season, and sun exposure were more significant predictors of serum 25(OH)D concentrations than dietary intake [30, 31].

The higher serum 25(OH)D concentrations in those who adhere more closely to a PD pattern may be related to certain lifestyle and health-related factors. In this study, we found that children with high PD pattern scores had consumed more supplements compared to this with high HFLVD pattern scores. Consumption of dietary supplements has been associated with an increase of serum 25(OH)D concentration [32]. Children who consumed vitamin D supplements were less likely to be vitamin D deficient [9]. Additionally, it has been suggested that the bioavailability of vitamin D may be low in those who are overweight or obese because of excessive sequestering of vitamin D in adipose tissue [33]. Gordon et al. [12] found that a higher BMI and being African American was associated with decreased serum 25(OH)D. Similarly, we found that subjects with high HFLVD pattern scores were tend to be overweight or obese and NHB. This could be a possible explanation of the lower serum 25(OH)D concentrations in those who adhered more closely to the HFLVD pattern. Furthermore, greater indoor activity measured by hours spent watching television, using computers, or playing video games has also been found to be a factor associated with lower 25(OH)D concentrations [9]. In the present study, there was a greater proportion of children who had ≤2 h/d of screen viewing time had higher PD pattern scores compared to those with high HFLVD pattern score suggesting decreased overall screen viewing time is an important factor for the increased serum 25(OH)D concentrations in those who adhere to a PD.

Furthermore, the relation of serum 25(OH)D with both dietary patterns remained for girls only in the adjusted analysis. Nanri et al. [34] found higher serum 25(OH)D concentrations in women who had higher fish/shellfish consumption and lower BMI. They proposed the difference may be related to body composition of females compared to males. Because women generally have higher fat mass than men and vitamin D is fat-soluble, this could result in higher amounts being stored in the fat tissue of females and lower serum vitamin D concentrations. This could be a possible explanation for why in the present study we have observed girls who adhere most closely to the HFLVD pattern have significantly lower serum vitamin D concentrations.

The present study has several strengths that included a nationally representative survey with a large sample size of children and adolescents. Because of a wide range of data are available on demographic characteristics, dietary information, and other health-related factors, we were able to adjust serum 25(OH)D concentrations for several known confounding variables. Results in this study can be interpreted towards the general US children and adolescents population because NHANES is based on a probability sample survey design and is representative of the US population. Because of cross-sectional nature of this study, cause and effect measurement is not possible. In addition, dietary intakes of children estimated by a FFQ may be underreported due to subjects’ inability to recall intakes accurately [35]. The errors in reporting food intakes may be minimal because the FFQ used in NHANES had been previously tested and validated by the National Cancer Institute.

## Conclusions

Overall, serum 25(OH)D concentration was associated with the PD pattern but not with the HFLVD pattern in US children and adolescents. When stratified by sex, the relation between dietary patterns and serum 25(OH)D was confined to only girls. Although vitamin D status has improved slightly recently, the hypovitaminosis D is still evident in US children and adolescents [36]. Because of hypovitaminosis D is linked to several chronic diseases [3–5], it is prudent to improve the vitamin D status of children to reduce the future risk for chronic diseases. In public health perspective, it is important to encourage children, especially girls who are consuming HFLVD pattern to shift to healthier diet.

## Abbreviations

25(OH)D: 25-hydroxyvitamin D
ANCOVA: Analysis of Covariance
BMI: Body mass index
FFQ: Food frequency questionnaire
H/MA: Hispanic/Mexican American
HFLVD: High Fat-Low-Vegetable Dietary Pattern
MEC: Mobile Examination Centers
NCHS: National Center for Health Statistics
NHANES: National Health and Nutrition Examination Survey
NHB: non-Hispanic black
NHW: non-Hispanic white
PD: Prudent Dietary Pattern
PIR: Poverty Income Ratio
SE: Standard error
T2DM: type-2 diabetes mellitus
